# Steamed *Panax notoginseng* Attenuates Anemia in Mice With Blood Deficiency Syndrome *via* Regulating Hematopoietic Factors and JAK-STAT Pathway

**DOI:** 10.3389/fphar.2019.01578

**Published:** 2020-01-21

**Authors:** Zejun Zhang, Yiming Zhang, Min Gao, Xiuming Cui, Yang Yang, Bert van Duijn, Mei Wang, Yupiao Hu, Chengxiao Wang, Yin Xiong

**Affiliations:** ^1^ Faculty of Life Science and Technology, Kunming University of Science and Technology, Kunming, China; ^2^ Institute of Biology Leiden, Leiden University, Leiden, Netherlands; ^3^ Fytagoras BV, Leiden, Netherlands; ^4^ LU-European Center for Chinese Medicine, Leiden University, Leiden, Netherlands; ^5^ SUBioMedicine BV, Leiden, Netherlands

**Keywords:** steamed *Panax notoginseng*, network pharmacology, JAK-STAT signaling pathway, anemia, blood deficiency syndrome, hematopoiesis

## Abstract

*Panax notoginseng* (Burk.) F. H. Chen is a medicinal herb used to treat blood disorders since ancient times, of which the steamed form exhibits the anti-anemia effect and acts with a “blood-tonifying” function according to traditional use. The present study aimed to investigate the anti-anemia effect and underlying mechanism of steamed *P. notoginseng* (SPN) on mice with blood deficiency syndrome induced by chemotherapy. Blood deficiency syndrome was induced in mice by cyclophosphamide and acetylphenylhydrazine. A number of peripheral blood cells and organs (liver, kidney, and spleen) coefficients were measured. The mRNA expression of hematopoietic function-related cytokines in the bone marrow of mice was detected by RT-qPCR. The janus kinase-signal transducer and activator of transcription (JAK-STAT) signaling pathway was screened based on our previous analysis by network pharmacology. The expression of related proteins and cell cycle factors predicted in the pathway was determined by Western blot and RT-qPCR. SPN could significantly increase the numbers of peripheral blood cells and reverse the enlargement of spleen in a dose-dependent manner. The quantities of related hematopoietic factors in bone marrow were also increased significantly after SPN administration. SPN was involved in the cell cycle reaction and activation of immune cells through the JAK-STAT pathway, which could promote the hematopoiesis.

## Introduction

Blood deficiency, a common syndrome seen in the clinic, is a pathological state of blood dysfunction and organs dystrophy according to the theory of traditional Chinese medicine (Li et al., 2015a). The diagnostic indicator of blood deficiency syndrome (BDS) mainly refers to the reduction of blood cells or hemoglobin, which is similar as anemia in modern medicine. Patients or animals with BDS often suffer from impaired hemopoietic function, peripheral blood pancytopenia, hypofunction of internal organs, malnutrition, or even myelosuppression during severe diseases (Zhang et al., 2014a; Ji et al., 2017). To find therapies to alleviate BDS and treat anemia has attracted tremendous attention in recent years. Hematopoietic cytokines, such as granulocyte-macrophage colony-stimulating factor (GM-CSF), interleukin-2 (IL-2), erythropoietin (EPO), and thrombopoietin (TPO), are sometimes used to accelerate the hemopoietic recovery during cancer therapy. However, due to the unstable efficacy, high cost, and adverse effects like osteomuscular pain, arthralgia, and allergic reactions (Dygai et al., 2012) caused by those treatments, the applications of above compounds regimens are limited.

It was reported that many Chinese herbal medicines with efficacies of enriching and regulating blood, could prevent and treat BDS as well as anemia (Li et al., 2012; Chen J. et al., 2019; Li et al., 2015b). *Panax notoginseng* (PN) Burk., a plant in genus *Panax* (Araliaceae), is a highly valued Chinese herbal medicine used to treat blood disorders in Asia for thousands of years (Wang et al., 2006). It was recorded in “Supplements for Compendium of Materia Medica” (1786) by Xue Min Zhao as such that PN root could be used as a hematinic drug (Guo et al., 2010). A steaming process is often used to enhance the “blood-tonifying” function of PN (Lau et al., 2003). According to our previous study, along with the duration of steaming, the levels of some major active saponins in raw PN were decreased and some other new saponins were produced or increased (Xiong et al., 2017a; Xiong et al., 2017b). Such transformation of active constituents in raw and steamed PN (SPN) contributes to the difference in their efficacies, such as that SPN is better at nourishing the blood and supplementing *qi* (vital energy) (The State Pharmacopoeia Commission of People's Republic, 2005; Xiong et al., 2017b). Although multiple pharmaceutic studies have been carried out to confirm the therapeutic effect of SPN on BDS (The State Pharmacopoeia Commission of People's Republic, 2005; Zhou et al., 2014; Xiong et al., 2017a; Xiong et al., 2017b), the underlying action mechanism is still unclear, which hinders the development of anti-anemia drugs from this herbal medicine of which side effects are only rarely reported.

To better elucidate the mechanism of SPN treating BDS, the classical BDS model induced by acetyl phenylhydrazine (APH) and cyclophosphamide (CY) was used in this study (Zhang et al., 2014a). Based on our previous analysis of the possible signaling pathways related to the hematinic effect of SPN using the network pharmacology, the janus kinase-signal transducer and activator of transcription (JAK-STAT) signaling pathway was chosen to further elucidate since it was predicted to be one of the pathways with a large number of potential targets involved (**Figure S1**) (Xiong et al., 2019). The pathway involves many members of the cytokine receptor superfamily, including GM-CSF, EPO, TPO, interferons, and numerous interleukins, which makes it central to hematopoietic cell biology and hematologic therapy alike (Darnell et al., 1994; Ward et al., 2000). In this study, based on the classical BDS mice model, the hematopoietic effect and working mechanism of SPN were investigated by evaluating the routine blood parameters, organ coefficients, and hematopoiesis-related factors involved in the JAK-STAT pathway. These data provide a theoretical basis for the use of SPN and its products in human health and provides clues for developing new drugs to treat BDS as well as anemia.

## Materials and Methods

### Preparation and Chemical Analysis of Steamed *Panax notoginseng*

Samples were collected from Wenshan, Yunnan in China (104°077ˊE, 23°188ˊN), which were identified to be the dried root and rhizome of *P. notoginseng* (Burkill) F. H. Chen by Prof. Xiuming Cui from Kunming University of Science and Technology. The specimen (No. WSPN15101) is deposited in Yunnan Key Laboratory of *P. notoginseng*, Kunming University of Science and Technology (**Figure S2**), which can be fully validated using http://mpns.kew.org/mpns-portal/?_ga=1.111763972.1427522246.1459077346. And the quality of samples was consistent with the requirements of Chinese Pharmacopoeia of 2015 edition (Chinese Pharmacopoeia Commission, 2015). The preparation of SPN was described in our previous study (Xiong et al., 2017b). In short, SPN samples were prepared by steaming the crushed raw materials in an autoclave (Shanghai Boxun Industry and Commerce Co., Ltd, China) for 6 h at 120°C. The steamed powder was then dried in a heating-air drying oven at about 45°C to constant weight, then powdered and sieved through a 40-mesh sieve.

Qualitative and quantitative analyses of SPN were performed as previous report (Xiong et al., 2017a). In brief, notoginsenoside R_1_ and ginsenosides Rg_1_, Re, 20(*R*)-Rh_1_, Rb_1_, Rd, Rk_3_, Rh_4_, 20(*S*)-Rg_3_, 20(*R*)-Rg_3_ (Sichuan Weikeqi Biological Technology Co., Ltd. Sichuan, China) with purity ≧ 98% were used as standard compounds. The analysis was performed on an Agilent 1260HPLC system (Agilent Technologies) equipped with a G1311B Pump, a G4212B DAD detector, and a G1329B autosampler. Chromatographic separation was carried out at 30°C on a Vision HT C_18_ column (250 × 4.6 mm, 5μm). The mobile phase was comprised of A (ultra pure water) and B (methyl cyanide). The gradient mode was as follows: 0–20 min, 80% A; 20–45 min, 54% A; 45–55 min, 45% A; 55–60 min, 45% A; 60–65 min, 100% B; 65–70 min, 80% A; 70–90 min, 80% A. The flow rate was set at 1.0 ml/min. The detection wavelength was set at 203 nm and sample volume was set at 10 μl. The HPLC chromatogram of SPN sample and chemical structures of dominating compounds characterized were shown in **Figure S3**.

### Animal

Animal experimental procedures in the study were strictly conform the Guide for the Care and Use of Laboratory Animals and related ethics regulations of Kunming University of Science and Technology. The protocol was approved by the Experimental Animal Welfare and Ethics Committee, Kunming University of Science and Technology. Sixty Kunming (KM) mice, half male and half female, weighing 18–22 g, were purchased from Tianqin Biotechnology Co. Ltd., Changsha, China [SCXK (Xiang) 2014-0011].

Mice were randomly divided into six groups, namely the control group, model group, *Fufang E'jiao Jiang* (FEJ) group, high-dose SPN (H-SPN) group, moderate-dose SPN (M-SPN) group, and low-dose SPN (L-SPN) group, 10 mice in each group (the power analysis to determine the sample size was given in **Table S1**). The experimental method was described in our previous study (Xiong et al., 2017a). “The BDS model was established by intraperitoneal injection of 0.07 g·kg^-1^ of CY for the first 3 days and a hypodermic injection of 0.02 g·kg^-1^ of APH on the fourth day. After that, mice in the control group were administered with 0.9% normal saline, whereas other groups were administered with FEJ (8 ml/kg^-1^), or SPN powder (1.8, 0.90, and 0.45 g·kg^-1^, respectively), respectively, by gavage for 12 days.”

### Blood Routine Test

Half an hour after the last experimental administration (saline, FEJ, or SPN), the mice were slightly anesthetized with a small amount of diethyl ether, and blood was taken from the orbit and collected into a sterile centrifuge tube containing sodium citrate. Blood was subjected to a peripheral hemogram analysis by a HEMAVET 950 full-automatic blood cell analyzer (DrewScientific Group, Dallas, TX, USA), for the quantification of red blood cells (RBCs), white blood cells (WBCs), hemoglobin (Hb) and platelets (PLTs).

### Organ Coefficient

After the anesthetization and blood collection as described in 2.3, the mice were sacrificed by cervical dislocation. The liver, kidney, and spleens were washed in normal saline solution, dried with filter paper, and weighed immediately. The spleens were stored in liquid nitrogen for Western blot analysis. Organ coefficient was calculated by the following equation: organ coefficient = [organ wet weight (g)/body weight (g)] × 100% (Zhang et al., 2014b).

### Analysis of EPO, EPO Receptor (EPOR), TPO, TPO Receptor (C-Mpl), GM-CSF, and GATA-1 in Mice Bone Marrow Nucleated Cells

One femur was isolated after the sacrifice of each mouse. The attached muscles were removed by gauze polishing. The femur was briefly soaked in 75% ethanol before washing with phosphate buffered solution for three times under aseptic conditions. The bone marrow cells were washed out of the femur with phosphate buffered solution several times and filtered with a 0.4 mm diameter needle. The bone marrow cell suspensions were separated with mice lymphocyte separation solution to prepare the bone marrow nucleated cells, after which 800 μl of RNAiso Plus kit (TaKaRa, Kusatsu, Japan) was added before storage at −80°C until real-time quantitative polymerase chain reaction (RT-qPCR) analysis was done. Total RNA from the bone marrow nucleated cells was extracted using the RNAiso Plus kit (TaKaRa, Kusatsu, Japan) in accordance with the manufacturer's protocols. Then total RNA (microgram) was reverse-transcribed into cDNA using a Primer-Script First Strand cDNA synthesis kit (TaKaRa, Kusatsu, Japan) (Yang et al., 2018). The design of gene-specific primers was done with Primer 5.0 software based on published mice sequences from the GeneBank/National Center for Biotechnology Information database and they were synthesized by TsingKe Biological Technology (Beijing, China). The nucleotide sequences primers used for PCR were shown in **Table 1**. The RT-qPCR program was conducted using a Light Cycler^®^ 96 System (DBI^®^ Bioscience, Ludwigshafen, Germany). Thermocycling parameters included an initial phase of 95°C for 2 min followed by 40 cycles of 95°C for 10 s and 60°C for 32 s. The relative gene expression values were calculated with the 2^−△△Ct^ method (Chen et al., 2017).

**Table 1:** Primers sequence and parameters for a reference gene and six target genes.

Gene	Gene ID	GenBank Accession	Primer sequence Forward/Reverse (5′-3′)	Product (bp)
EPO	13856	NC_000071.6	F: TGGAGGTGGAAGAACAGG R: GCAGTGAAGTGAGGCTACG	165
EPOR	13857	NC_000075.6	F: CGCTTGGAAGACTTGGTGTGT R: CTCACCCTCGAGCTGGTATGA	90
TPO	22018	NC_000078.6	F: CCAGACGGAACAGAGCAAG R: CTGTCCTCGTGCTGCCAT	82
c-Mpl	17480	NC_000070.6	F: CCTGCACTGGAGGGAGGTCT R: GGCTCCAGCACCTTCCAGTC	135
GM-CSF	12981	NC_000077.6	F: AAGCCCTAAACCTCCTGGATG R: GCCTACCAGACATACTGCCCCCC	142
GATA-1	14460	NC_000086.7	F: GGAGGGACAGGACAGGTCACT R: GTTTGCTGACAATCATTCGCTT	110
β-actin	11461	NC_000071.6	F: CCAGCCTTCCTTCTTGGGTAT R: CATAGAGGTCTTTACGGATGTCAAC	97

### Pathway and Targets Analyses

#### Prediction

Based on previous research (Xiong et al., 2019), 20 compounds including ginsenosides of F_2_, Rb_1_, Rb_2_, Rb_3_, Rc, Rd, Re, Rg_1_, Rh_2_, Rh_4_, Rk_3_, 20(*R*)-Rg_2_, 20(*S*)-Rg_2_, 20(*R*)-Rg_3_, 20(*S*)-Rg_3_, 20(*R*)-Rh_1_, and 20(*S*)-Rh_1_; and notoginsenosides of C, R_1_, and R_2_ reported in SPN were selected to construct the network. “The chemical structures of the composite compounds in SPN were obtained from traditional Chinese medicine Database@Taiwan (TDT) or drawn with ChemDraw professional 15.0 (Chen, 2011). The targets of constituents were predicted by the online target prediction software of Pharmmapper with a criterion of “fit score” > 4 (http://lilab.ecust.edu.cn/pharmmapper/index.php) (Wang et al., 2017). Gene and protein targets associated with the disease of anemia were collected from the Online Mendelian Inheritance in Man (OMIM) database (Amberger et al., 2015). Database of Interacting Proteins for protein-protein interactions (PPI) was employed to identify the possible interactions of the aforementioned targets. All protein ID codes were converted to UniProt IDs (Wei et al., 2016).” We used the DAVID (a Database for Annotation, Visualization, and Integrated Discovery) Functional Annotation Bioinformatics Microarray Analysis (https://david.ncifcrf.gov/) for pathway enrichment analysis and the Kyoto Encyclopedia of Genes and Genomes (KEGG) to construct the “SPN components-target-anemia” network with protein-protein interactions information. Three topological parameters of “degree,” “betweenness centrality” and “close centrality,” and Cytoscape 4.3 were applied to screen potential targets for SPN treating anemia (Xiong et al., 2019). These relevant proteins were put into DAVID to perform gene ontology terms and KEGG pathway analysis.

#### Verification

##### Total Protein Extraction and Western Blotting Analysis

Spleen proteins were extracted using RIPA lysis buffer (Solarbio, Beijing, China) and centrifuged at 12,000 g for 10 min at 4°C. Protein concentration was assessed using the BCA Protein Assay Kit (Biosharp, Beijing, China). Samples containing equal amounts of protein (80 μg) were separated by 10% sodium dodecyl sulfate-polyacrylamide gel electrophoresis and then transferred to 0.45 μm polyvinylidene difluoride membranes (Biosharp, Beijing, China). The non-specific binding sites on the membrane were then blocked with 5% fresh non-fat dry milk in Tris buffered saline TBS with Tween (10 mM Tris, 150 mM NaCl, pH7.4) with 0.1% Tween 20) for 2 h. Subsequently, they were incubated with primary antibodies (anti-IL-2, anti-STAT1, anti-SHP2, anti-JAK1, and anti-p-JAK1; 1:1000 dilution; Abcam, Cambridge, UK) and primary β-actin antibody (1:1,000 dilution; abmart, Shanghai, China) overnight at 4°C, respectively. After this, the membrane was washed followed by three 10 min washes in TBST, and then incubated with anti−mouse secondary antibody (1:2000 dilution; Cell Signaling Technology, Shanghai, China) for 2 h at room temperature followed by three 10 min washes in TTBS. Finally, the chemiluminescent signals were observed with a multifunctional gel imaging system (Bio-Rad, USA) and densitometry for immunoreactive bands was performed with National Institutes of Health software (Image J).

##### Analysis of IL-2, STAT1, SHP2, p-JAK1, Bcl-2, Bcl-XL, c-Myc, and p21 in Mice Spleen by RT-qPCR

The analysis method refers to item 2.5. IL-2, STAT1, SHP2, p-JAK1, Bcl-2, Bcl-XL, c-Myc, and p21 genes sequence synthesized by TsingKe Biological Technology (Beijing, China), and primers sequence are shown in **Table 2**.

**Table 2:** Primers sequence and parameters for IL-2, STAT1, SHP2, p-JAK1, Bcl-2, Bcl-XL, c-Myc, and p21 genes.

Gene	Gene ID	GenBank Accession	Primer sequence Forward/Reverse (5′-3′)	Product (bp)
IL-2	16183	NC_000069.6	F: CCTGAGCAGGATGGAGAATTACA R: TCCAGAACATGCCGCAGAG	141
STAT1	20846	NC_000067.6	F: AAACTGCCAACTCAACACCTCT R: AGACCACCTCTCTTCCTGTCGT	107
SHP2	19247	NC_000071.6	F: ATCCGCCAGAAGTCATTCACC R: TTTGATCATACCAGGGTCGTT	178
p-JAK1	16451	NC_000070.6	F: TGGAGGTAACCACATAGC R: CCGAGAACCCAAATAGTC	250
Bcl-2	12043	NC_000067.6	F: CCTGTGCCACCATGTGTCCATCC R: GCTGAGAACAGGGTCTTCAGAGAC	400
Bcl-XL	12125	NC_000068.7	F: TGGATCTCTACGGGAACAATGC R: GTGGCTGAAGAGAGAGTTGTGG	197
c-Myc	17869	NC_000081.6	F: TCAAGAGGTGCCACGTCTCC R: TCTTGGCAGCAGGATAGTCCTT	81
p21	12575	NC_000083.6	F: GTGTGCCGTTGTCTCTTCGG R: CTCAGGTAGACCTTGGGCAG	188

### Statistical Analyses

All data are expressed as means ± standard deviation. SPSS 21.0 software (Statistical Program for Social Sciences, SPSS Inc, USA) was applied to carry out the two-tailed unpaired *t*-test. A value of *P* < 0.05 was considered to indicate a significant difference. A value of *P* < 0.01 was considered to indicate a highly significant difference. Quantitative gene expressions were determined by RT-qPCR and histograms were drawn by GraphPad Prism 7 software (GraphPad Software, USA).

## Results

### General Behavior of Mice

The change of general behavior of mice can reflect the occurrence and recovery of BDS of mice to certain degree (Li et al., 2012). After the induction of BDS, mice in the model group became sluggish, exhausted and somnolent, along with weight loss, sparse hair, pale ears and tail, and loss of appetite. Those symptoms were consistent with the description of BDS in Chinese medicine (Ji et al., 2017). In contrast, mice in the control group and BDS-induced mice treated with FEJ or SPN were relatively vigorous and strong, presenting thick and lustrous hair, pink and moist nose and lips, round and pink tail, gained weight and better appetite.

### Blood Routine Test

**Figure 1:**
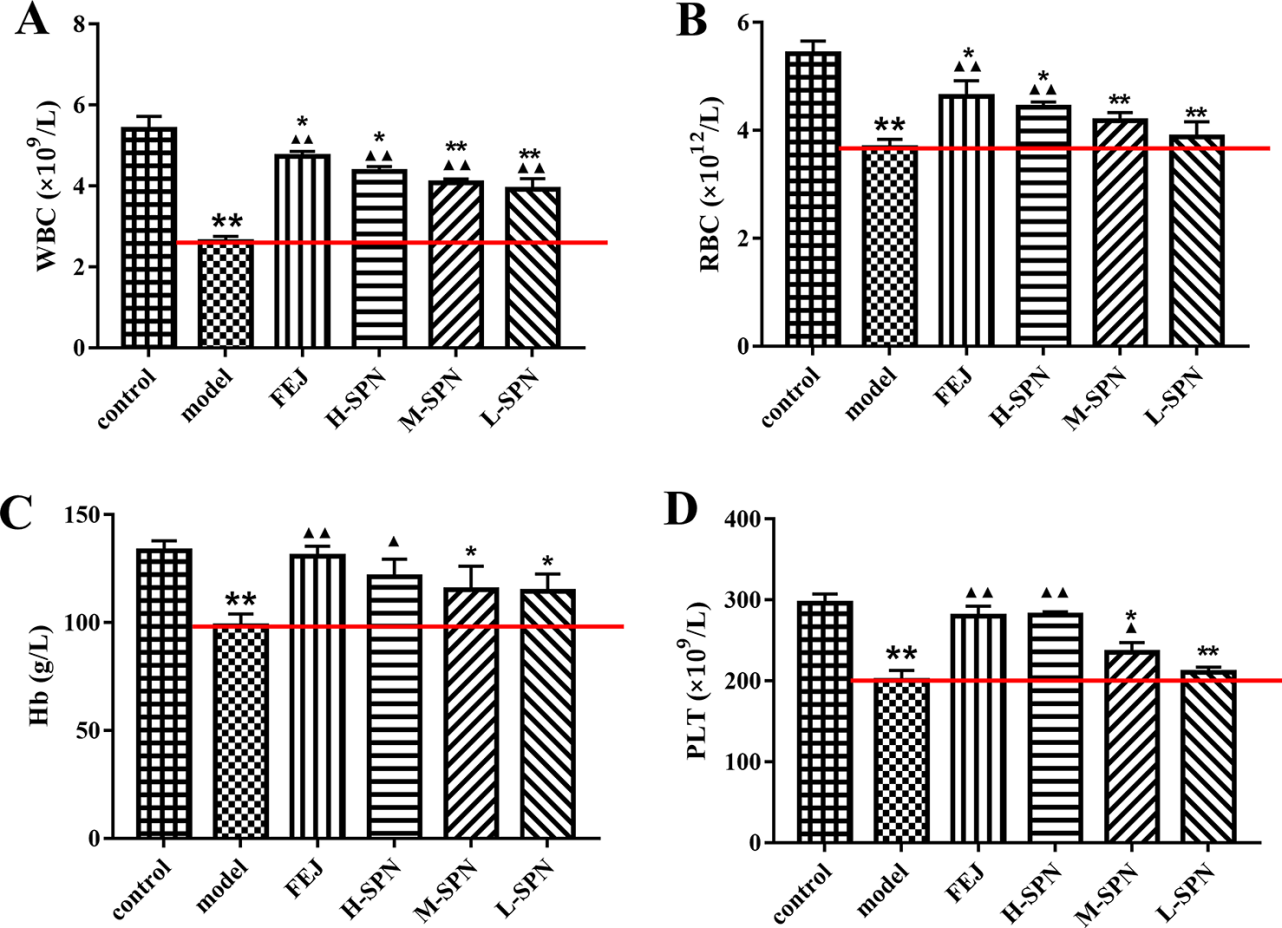
The blood parameters of the control and model mice after treatment with saline solution (model), FEJ and different amounts doses of SPN. **(A)** The number of WBC. **(B)** The number of RBC. **(C)** The content of Hb. **(D)** The number of PLT. Each value represents the mean ± SD (n = 10); *p < 0.05 and **p < 0.01, compared to the control group; ^▲^ *p* < 0.05 and ^▲▲^ *p* < 0.01, compared to the model group.

After the administration of saline, FEJ or SPN for 12 days, the quantities of WBC, RBC, Hb, and PLT from the peripheral blood of mice were determined (**Figure 1**). Compared to the control group, the levels of WBC, RBC, Hb, and PLT in the model group were decreased significantly (*p* < 0.01), indicating the anemia model was established successfully. Comparison within the model group shows that WBC, RBC, Hb and PLT levels in mice treated with FEJ and H-SPN were increased significantly (*p* < 0.01 or *p* < 0.05). In particular, all three doses (H, M, and L) of SPN significantly improved the level of WBC (*p* < 0.01). RBC and Hb levels in mice treated with M-SPN and L-SPN were not significantly increased.

### Organ Coefficients

**Figure 2:**
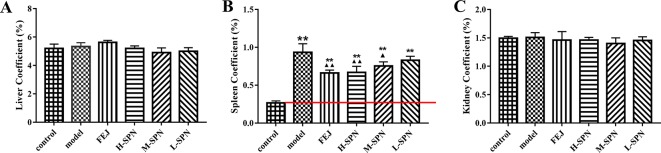
The organ coefficients of control and model mice after treatment with saline solution (model), FEJ and different doses of SPN. **(A)** The liver coefficient. **(B)** The spleen coefficient. **(C)** The kidney coefficient. Each value represents the mean ± SD (n = 10); **p < 0.01, compared to the control group; ^▲^ *p* < 0.05 and ^▲▲^ *p* < 0.01, compared to the model group.

The results for organ coefficients are shown in **Figure 2**. Compared to the control group, the liver and kidney of the model group were relatively small, but the spleen was abnormally enlarged. Except for the spleen coefficient, there was no significant difference in other organ coefficients between the experimental groups (model, FEJ, H-SPN, M-SPN, and L-SPN group) and the control group. Compared to the control group, the spleen coefficient of the model group was increased significantly (*p* < 0.01). Comparison within the model group shows that the spleen coefficients of the FEJ and H-SPN groups were decreased significantly (*p* < 0.01), the M-SPN group was decreased significantly (*p* < 0.05), whereas there was no significant difference for the L-SPN group. According the results, SPN induces a reversing effect on the enlargement of spleen in a dose-dependent way.

### Effect of SPN on EPO, EPOR, TPO, c-Mpl, GM-CSF, and GATA-1 in Mice Bone Marrow

**Figure 3:**
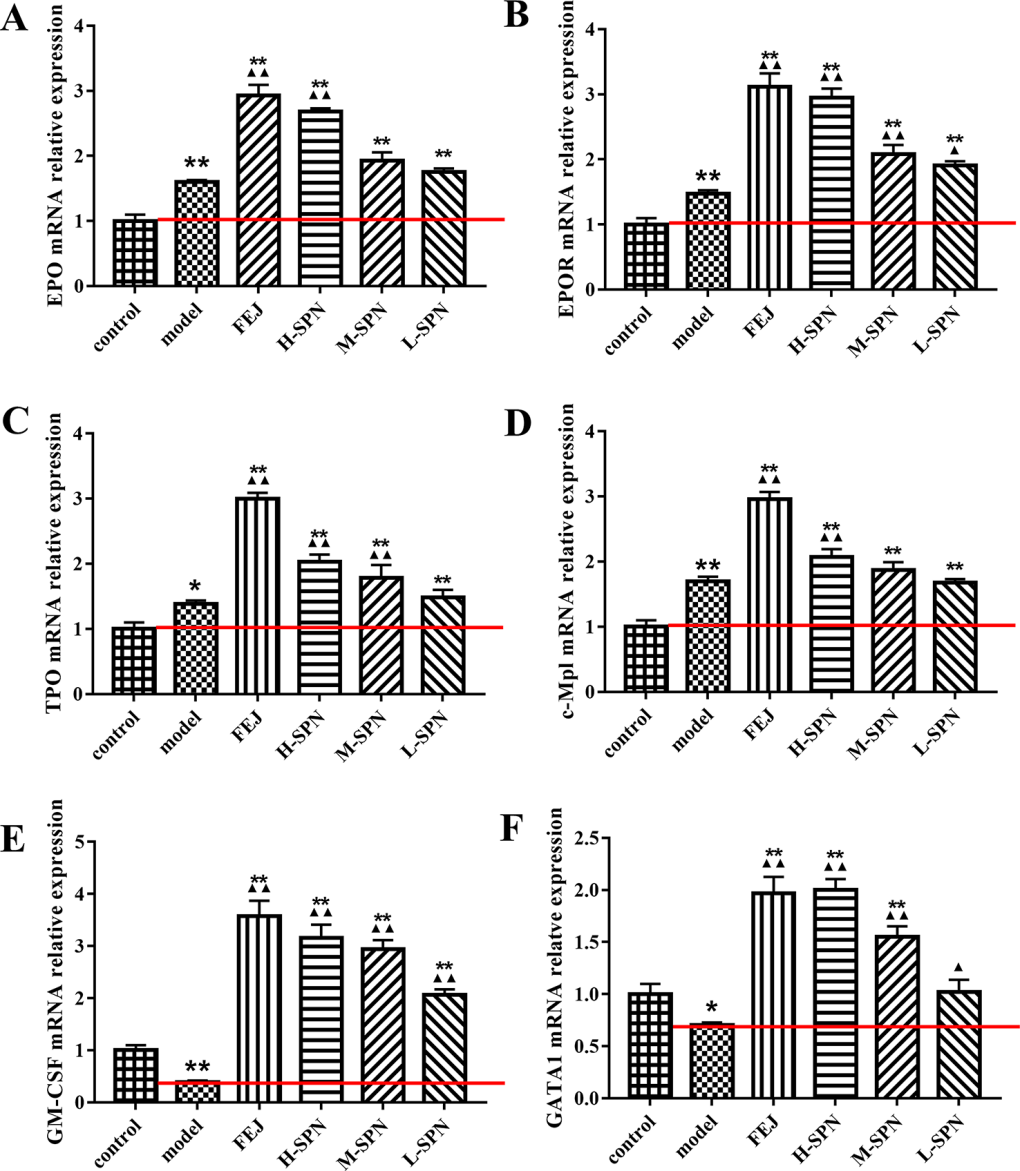
The mRNA expression of **(A)** EPO, **(B)** EPOR, **(C)** TPO, **(D)** c-Mpl, **(E)** GM-CSF, and **(F)** GATA1 in control and model mice after treatment with saline solution (model), FEJ and different doses of SPN. Each value represents the mean ± SD (n = 10); *p < 0.05 and **p < 0.01, compared to the control group; ^▲^ *p* < 0.05 and ^▲▲^ *p* < 0.01, compared to the model group.

The effects of FEJ and SPN on the mRNA expression of hematopoietic function-related cytokines in the bone marrow of mice is shown in **Figure 3**. Compared to the control group, the levels of EPO, EPOR, TPO and c-Mpl in the model group were increased significantly (*p* < 0.01 or *p* < 0.05). Comparison within model group shows that the levels of the above cytokines in mice treated with FEJ and H-SPN were increased significantly (*p* < 0.01). Increases to different degrees were also seen in the M-SPN and L-SPN groups. Compared to the control group, the levels of GM-CSF and GATA1 in the model group were decreased significantly (*p* < 0.01 or *p* < 0.05). Comparison within the model group shows that both cytokines were significantly increased in mice treated with FEJ, H-SPN, M-SPN and L-SPN (*p* < 0.01 or *p* < 0.05). The mRNA expression levels of these hematopoietic related cytokines were changed in a SPN dose-dependent manner.

### Pathway and Targets Analyses

#### Prediction

**Figure 4:**
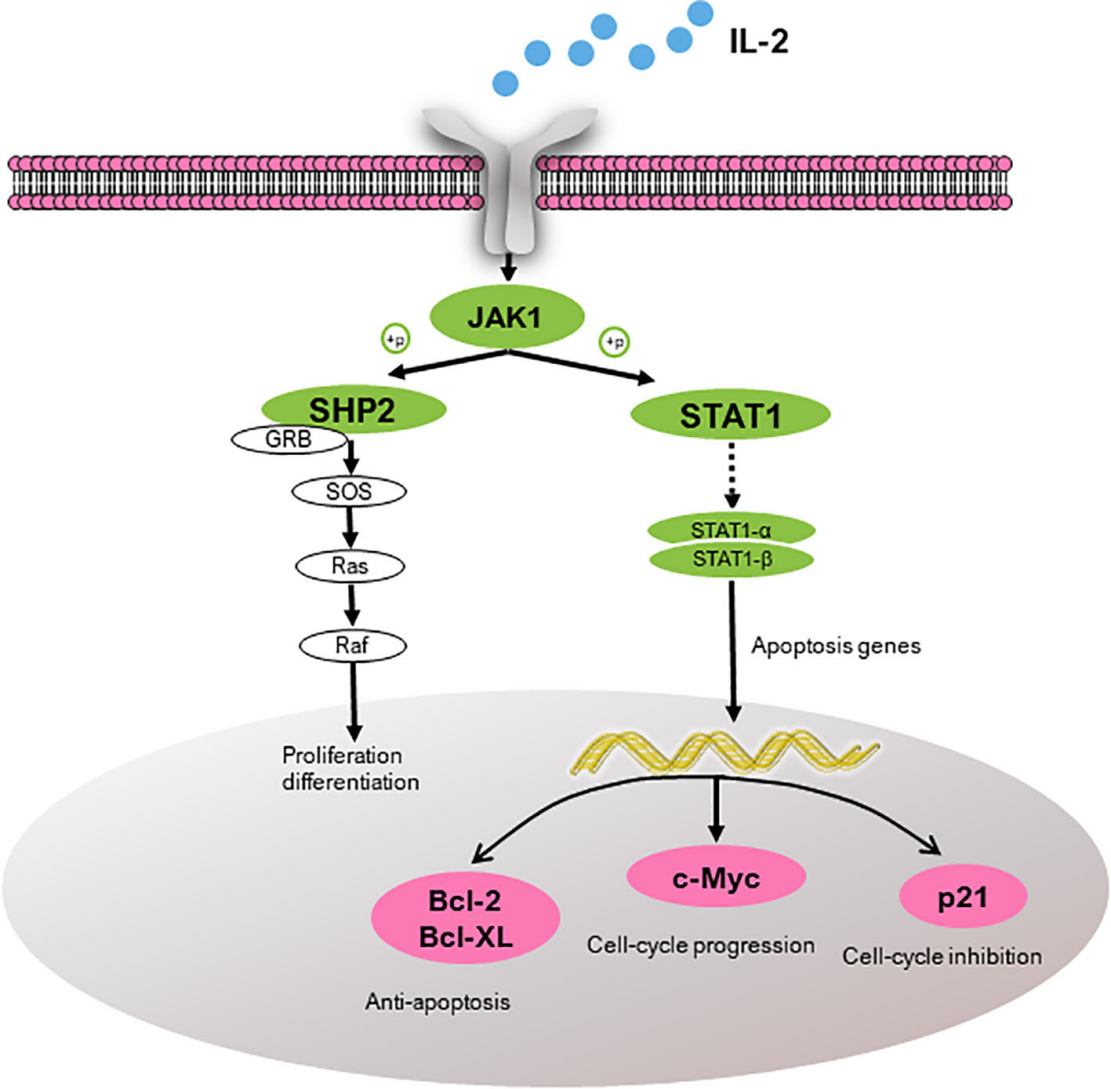
Target proteins (IL-2, SHP2, and STAT1) predicted to be involved in the JAK-STAT pathway and the downstream cytokines (Bcl-2, Bcl-XL, c-Myc, and p21). IL-2, interleukin-2; JAK1, janus kinase-1; SHP2, tyrosine-protein phosphatase non-receptor type 11; GRB, growth factor receptor-bound protein; SOS, guanine nucleotide exchange factor; STAT1, signal transducer and activator of transcription; Bcl-2, B-cell lymphoma-2; Bcl-XL, Bcl-2 like 1; c-Myc, v-myc avian myelocytomatosis viral oncogene homolog; and p21, cki.

According to the pathway enrichment analysis, the JAK-STAT signaling pathway was predicted to be one of the most enriched pathways (**Figure S1**). Based on the KEGG analysis, three anemia-related targets are involved in the pathway and corresponding active constituents in PN are predicted and shown in **Table 3** (Xiong et al., 2019). Another kinase protein, JAK1, between IL-2 and STAT1 or SHP2, also plays a significant pivotal role in the JAK-STAT pathway (**Figure 4**). Therefore, the expression of IL-2, JAK1, p-JAK1, and STAT1 proteins in BDS mice was investigated in the following analysis. In addition, the mRNA expression of downstream cytokines including Bcl-2, Bcl-XL, c-Myc, and p21 related to the cell cycle was investigated based on the prediction and reference studies (Fröjmark et al., 2010; Karimian et al., 2016).

**Table 3:** The information of three targets and their corresponding active constituents involved in JAK-STAT signal pathway predicted by KEGG.

Gene ontology terms	Protein name	Abbreviations	Closeness centrality	Degree	Betweeness centrality	Constituent predicted
P60568	Interleukin-2	IL-2	0.2404	4	0.0056	Ginsenosides Rb_2_ and 20(*R*)-Rg_3_
Q06124	Tyrosine-protein phosphatase non-receptor type 11	SHP2	0.2812	6	0.0078	Ginsenosides Rd, Rh_4_, and Rk_3_
P42224	Signal transducer and activator of transcription 1-alpha/beta	STAT1-α/β	0.2188	2	0.0024	Notoginsenoside C

#### Western Blotting Analysis

**Figure 5:**
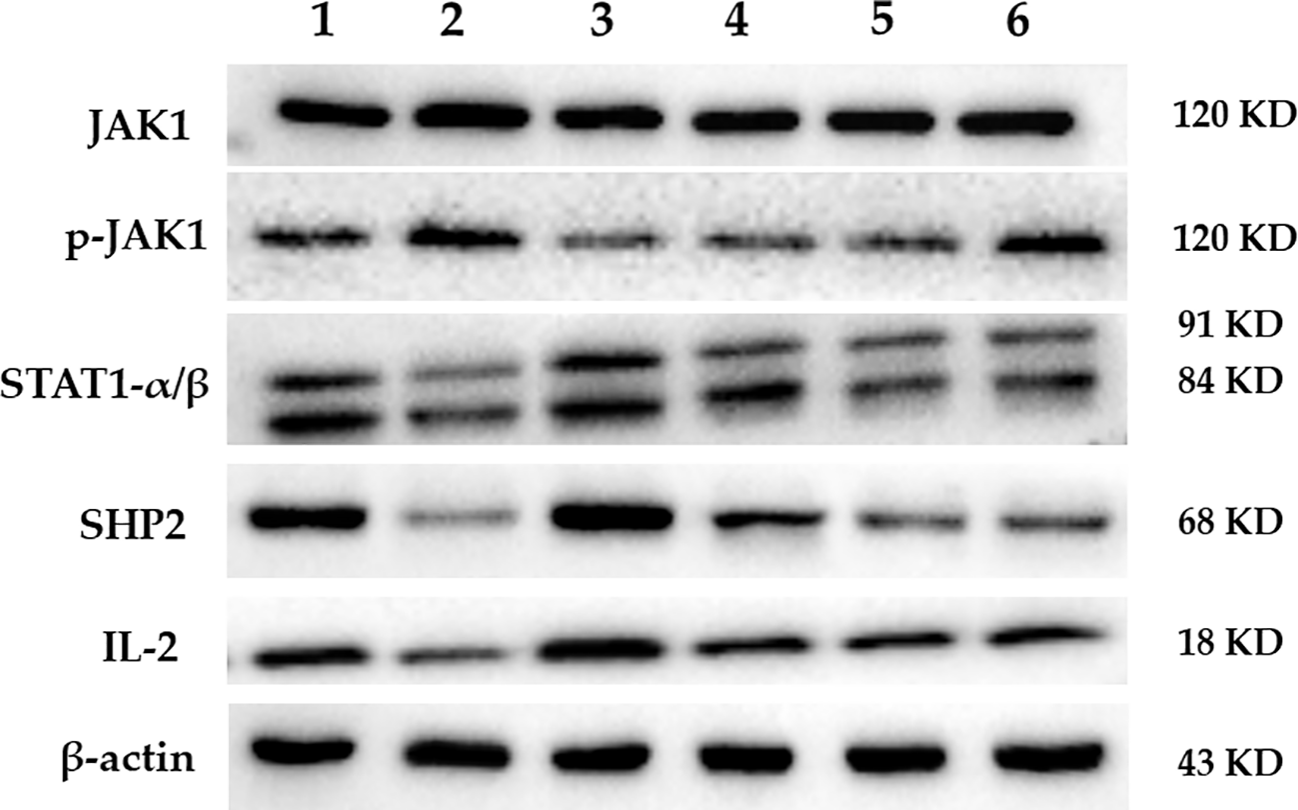
The expression of predicted target protein by Western blot. Lanes 1–6 represent control, model, FEJ, H-SPN, M-SPN, and L-SPN group, respectively.

The protein expression of JAK1, p-JAK1, STAT1, SHP2, and IL-2 in the spleen of mice in each group is shown in **Figure 5**. The results of the gray scale analysis are shown in **Table 4**. According to the results, there was no significant difference in JAK1 expression among the groups (*p* > 0.05). The expression level of p-JAK1 in the model group was significantly higher than in the control group (*p* < 0.01). Both FEJ, H-, and M-SPN result in significantly lower protein expression levels than in the model group (*p* < 0.01). Compared to the control group, the expression levels of protein STAT1, SHP2 and IL-2 in the model group were significantly decreased (*p* < 0.01 or *p* < 0.05). Comparison within the model group shows that the expression levels of the protein IL-2 after treatment with FEJ, H-SPN, M-SPN or L-SPN were significantly increased (*p* < 0.01 or *p* < 0.05). The expression levels of the protein STAT1 and SHP2 in FEJ and H-SPN groups were significantly higher than those in the model group, and the differences were statistically significant (*p* < 0.01 or *p* < 0.05), while those for in M-SPN and L-SPN groups were not statistically significant (*p* > 0.05).

**Table 4:** Gray value ratio of predicted target proteins/β-actin by Western blot.

	JAK1	p-JAK1	STAT1	SHP2	IL-2
Control	1.821 ± 0.0761	1.0237 ± 0.0563	1.1813 ± 0.1527	1.7962 ± 0.137	1.5006 ± 0.08375
Model	1.8976 ± 0.0921	1.2527 ± 0.0469**	0.7504 ± 0.0973*	0.5483 ± 0.0937**	1.07862 ± 0.0297*
FEJ	1.9013 ± 0.0631	0.823 ± 0.731▲▲	1.2875 ± 0.1268▲	1.9767 ± 0.1403▲▲	1.7025 ± 0.085▲▲
H-SPN	1.8356 ± 0.0821	0.9651 ± 0.3758▲▲	0.8653 ± 0.1348▲	1.1301 ± 0.1335▲	1.2779 ± 0.0574▲
M-SPN	1.8445 ± 0.0824	1.0014 ± 0.5728▲▲	0.6993 ± 0.0838	1.0099 ± 0.061	1.2375 ± 0.0543▲
L-SPN	1.8713 ± 0.0762	1.2352 ± 0.3862	0.7571 ± 0.1019	1.0894 ± 0.0208	1.2104 ± 0.066▲

Each value represents the mean ± SD (n = 10); *p < 0.05 and **p < 0.01, compared with the control group; ^▲^p < 0.05 and ^▲▲^p < 0.01, compared with the model group.

#### Quantification of IL-2, STAT1, SHP2 and p-JAK1 by RT-qPCR in Mice Spleen

**Figure 6:**
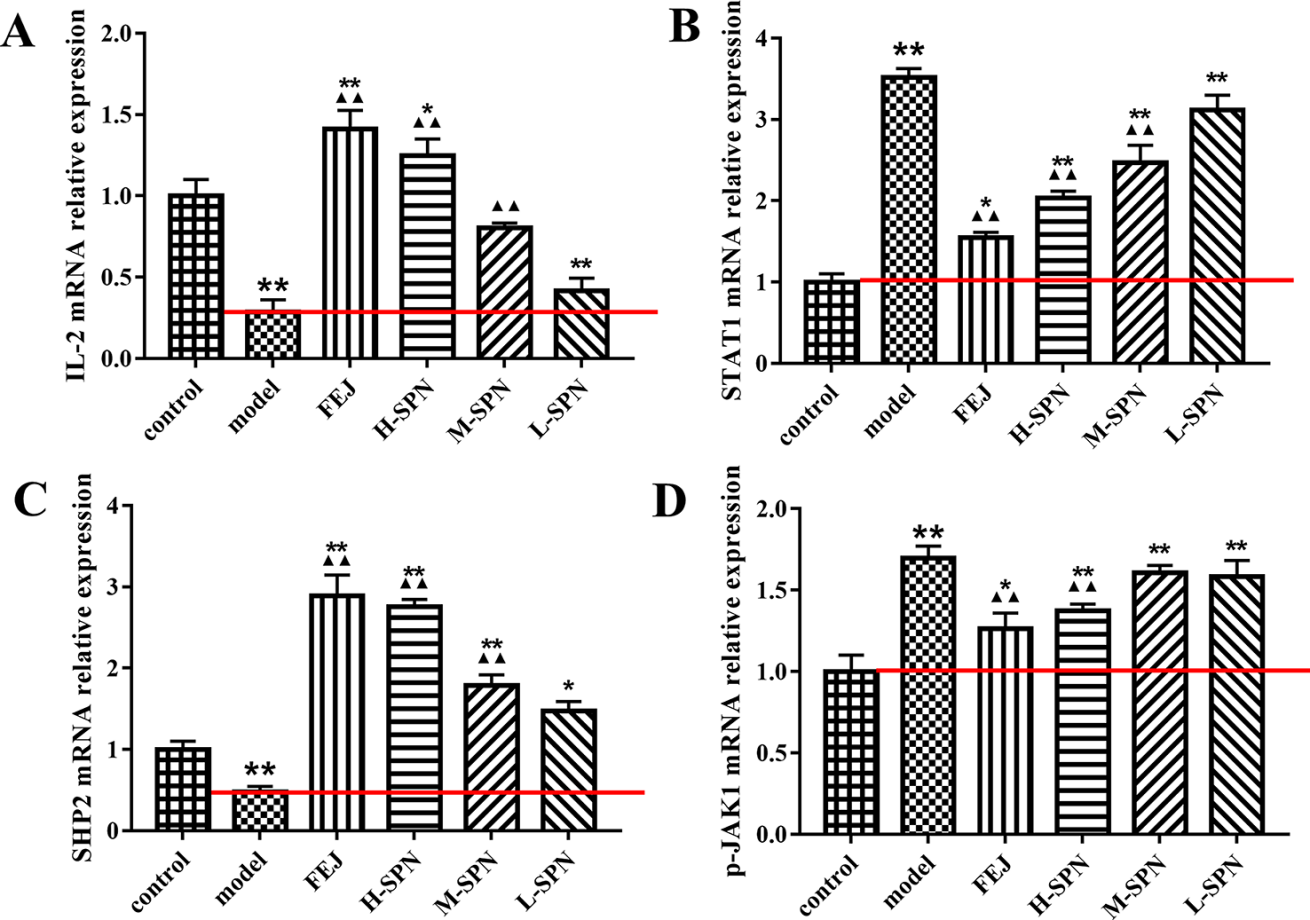
The mRNA expression of **(A)** IL-2, **(B)** STAT1, **(C)** SHP2, and **(D)** p-JAK1 in control and model mice after treatment with saline solution (model), FEJ and different doses of SPN. Each value represents the mean ± SD (*n* = 10); *p < 0.05 and **p < 0.01, compared to the control group; ^▲^ *p* < 0.05 and ^▲▲^ *p* < 0.01, compared to the model group.

The expression of IL-2, STAT1, SHP2 and p-JAK1 mRNA in the spleen of mice is shown in **Figure 6**. Compared to the control group, the expression of IL-2 and SHP2 mRNA in the model group was significantly down-regulated (*p* < 0.01). Comparison within the model group shows that after treatment with FEJ or different dose of SPN, the expression of IL-2 and SHP2 mRNA in the administered groups was increased to varying degrees in a dose-dependent manner. There was no significant difference in the expression of IL-2 and SHP2 between the L-SPN group and the model group (*p* > 0.05), others are statistically significant (*p* < 0.01). Compared to the control group, the expression of STAT1 and p-JAK1 mRNA in the model group was significantly up-regulated (*p* < 0.01). Comparison within the model group shows that after treatment with FEJ or different dose of SPN, the expression of STAT1 and p-JAK1 mRNA in the administered groups was decreased and negatively related to the drug concentration. There was no significant difference in the expression of STAT1 and p-JAK1 between the L-SPN group and the model group (*p* > 0. 05). There was no significant difference in the expression of p-JAK1 between the M-SPN group and the model group (*p* > 0. 05). And there was significant difference between the other groups (*p* < 0. 01).

#### Quantification of JAK1-STAT1 Downstream Cytokines in Mice Spleen by RT-qPCR

**Figure 7:**
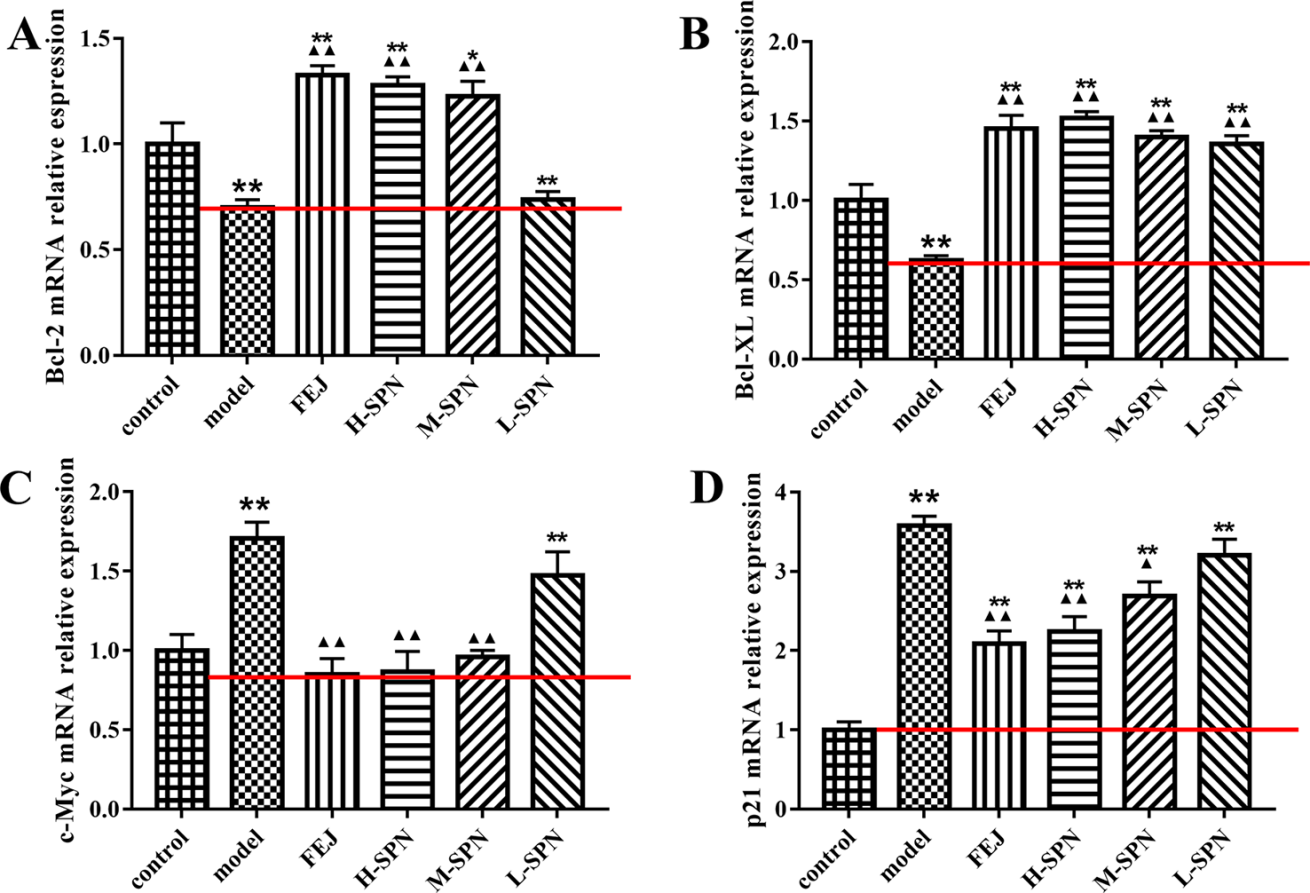
The mRNA expression of **(A)** Bcl-2, **(B)** Bcl-XL, **(C)** c-Myc, and **(D)** p21 in control and model mice after treatment with saline solution (model), FEJ and different doses of SPN. Each value represents the mean ± SD (*n* = 10); *P < 0.05 and **P < 0.01, compared to the control group; ^▲^ *P* < 0.05 and ^▲▲^ *P* < 0.01, compared to the model group.

The expression of Bcl-2, Bcl-XL, c-Myc, and p21 mRNA in the spleen of mice is shown in **Figure 7**. Compared to the control group, the expression of Bcl-2 and Bcl-XL mRNA in the model group was highly significantly down-regulated (P < 0.01). Comparison within the model group shows that after treatment with FEJ or different dose of SPN, the expression of Bcl-2 and Bcl-XL mRNA in the administered group was increased to varying degrees in a dose-dependent manner. Except that there was no significant difference between the Bcl-2 mRNA of L-SPN and the model group (*p* > 0.05), and the other groups were statistically significant (*p* < 0.01). Compared to the control group, the expression of c-Myc and p21 mRNA in the model group was highly significantly up-regulated (*p* < 0.01). Comparison within the model group shows that after treatment with FEJ or different dose of SPN, the expression of c-Myc and p21 mRNA in the administered group decreased and was negatively related to the drug concentration. Except that there was no significant difference between the L-SPN and the model group (*p* > 0.05), and the other groups were statistically significant (*p* < 0.01).

## Discussion

According to traditional Chinese medicine theory, the pathogenesis of blood deficiency could be the disharmony of *Yin-Yang*, *qi* deficiency, blood stasis, and the dysfunction of internal organs. Therefore, herbal medicines which can nourish *qi* and blood, as well as invigorate the spleen and kidney are often used in the clinic to treat blood deficiency and anemia (Hijikata et al., 2009; Wu et al., 2013; Sun et al., 2016). For example, FEJ, the positive control in the study, is a widely used immune-boosting traditional Chinese formula which has been clearly confirmed to promote the recovery of bone marrow hemopoietic activity and enhance the immune function (Liu et al., 2014; Shen et al., 2016). While for SPN, another tonic and hemostatic drug used for more than 400 years, the possible mechanism of the “blood-tonifying” effect is unclear yet. After the modeling, there was an obvious change in the appearance of mice, including hair loss, movement retardation, and weight loss, with the significant decrease in the peripheral WBC, RBC, Hb, and PLT, which are characteristic clinical manifestations of blood deficiency as well as anemia (Sun et al., 2016). With the H-SPN and FEJ treatments, the decrease of peripheral blood cells was reversed significantly (**Figure 1**), suggesting that H-SPN and FEJ could enhance the hematopoietic effect of mice with BDS, consistent with the traditional use of SPN and FEJ to tonify the blood (Gu et al., 2015). Meanwhile, M-SPN and L-SPN could not increase the numbers of RBC, Hb and PLT significantly, suggesting that a higher dosage of SPN was required to achieve a more obvious therapeutic effect, which was consistent with our previous study (Xiong et al., 2017a). Another diagnostic consideration for anemia is the enlarged size of spleen, which is one of the most common and early organs to be affected in different types of anemia (Dhaliwal et al., 2004; Alsalem, 2011). It is generally accepted that the hematopoietic deficiency is associated to a massive migration of bone marrow progenitor cells out of the marrow, *via* the blood, leading to an accumulation in the spleen (Queiroz et al., 2004). And the pooling of RBCs in the enlarged spleen can exacerbate the syndrome of anemia in turn (Gaspar et al., 2015). Therefore, the decline of RBCs in circulation and anemia can be both correlated with the extent of the enlargement of the spleen (Wagner et al., 2000). According to **Figure 3**, the weight and size of spleen in the model group were increased. While the spleen of mice treated with FEJ, H-SPN, and M-SPN was kept from enlarging compared to the model group (**Figure 2C**). It indicates that FEJ, H-SPN, and M-SPN could alleviate the side effects of chemotherapy on the spleen and protect the immune organ.

The process of blood cell formation, by which a small number of self-renewing stem cells give rise to lineage committed progenitor cells that subsequently proliferate and differentiate to produce the circulating mature blood cells, is regulated by a series of related hematopoietic related cytokines (Sieff, 1987). Hematopoietic growth factors (HGFs) including EPO, TPO, GM-CSF, and IL-3 are cytokines involved in the regulation of hematopoiesis (Ferretti et al., 2006; Genova et al., 2016). Among them, EPO is a glycoprotein hormone and a major agonist for the production of WBC (Franke et al., 2013). EPO binds to its receptor EPOR, activates the downstream JAK-STAT signaling pathway, promotes the growth and differentiation of erythroid progenitor cells, inhibits their apoptosis, and mobilizes hematopoietic stem cells (HSCs) (Madonna et al., 2009; Elliott et al., 2014). The results in **Figures 3A**, B show that the expressions of EPO and EPOR mRNA in the model group was higher than those in the control group, suggesting that the chemotherapy-induced anemia caused a feedback increase in EPO content, and then induced the expression of EPOR. This is a compensatory mechanism. Due to the damage of chemotherapy drugs to bone marrow cells, the reactivity of reactive cells to HGFs is reduced. So a higher concentration of HGFs is required to promote the recovery of hematopoietic function, which is consistent with the above literature reports. While after treating with the drugs, the expression of EPO and EPOR mRNA in the administration groups (FEJ, H-SPN, M-SPN, and L-SPN groups) was higher than that in model group, indicating that FEJ and SPN could promote the recovery of erythropoiesis by promoting the synthesis and secretion of EPO in mice bone marrow. A similar tendency was also observed in the level change of TPO and its receptor c-Mpl in the bone marrow of mice (**Figures 3C, D**), the former of which is the most important regulator of platelet production, as well as a major cytokine required for the development of HSCs, monocytes, granulocytes, mast cells and dendritic cells (Chen, 2004; Kirshenbaum et al., 2004). After administration of FEJ and SPN, the expression of TPO and c-Mpl mRNA in bone marrow was significantly increased, which would be beneficial to promote the recovery of megakaryocytes hematopoiesis and increase the number of peripheral PLT in mice with BDS. Another important HGF is GM-CSF, which mainly acts on HSCs/HPCs and bone marrow stromal cells or fibroblasts, and plays an important role in regulating hematopoiesis and leukocyte formation (Kimura et al., 1997; Waller, 2007). The results in **Figure 3E** showed that APH and CY significantly inhibited the expression of GM-CSF mRNA, which was increased significantly after the administration of FEJ and SPN. It indicated that FEJ and SPN could inhibit the cytotoxicity of CY and APH to some extent. GM-CSF level was induced to increase, promoting the recovery of granulocytic hematopoietic function. We also investigated the change of GATA1, a necessary transcription factor for the development and maturation of normal erythrocytes. The increased expression of GATA1 mRNA after treating FEJ and SPN was consistent with the above mentioned cytokines (**Figure 3F**).

From the results of responses of the hematinic function-related cytokines, we noted that the expression of them were closely related to the signal pathway of JAK-STAT, which plays a critical role in transduction of extracellular signals from cytokines and growth factors involved in hematopoiesis, immune regulation and inflammation (Vainchenker and Constantinescu, 2013; Chen et al., 2016). According to our previous analysis of network pharmacology, JAK-STAT was also predicted to be one of the major pathways related to the treatment of anemia with SPN (**Figure S1**). IL-2, STAT 1, and SHP2 in **Figure 4**, were predicted to be the major three targets involved in the JAK-STAT pathway to treat anemia (Xiong et al., 2019). To verify the prediction, we investigated the effect of SPN on the expression of the three targets and also the downstream cytokines of JAK-STAT including Bcl-2, Bcl-XL, c-Myc, and p21. According to the results, the treatment by APH and CY could significantly inhibit the expression of IL-2 and SHP2 proteins (**Figure 5**) and the corresponded mRNA's (**Figures 6A, C**). Since IL-2 is a Th1 type cytokine secreted by T cells (Vafadari et al., 2012) and SHP2 plays an up-regulation role in the development and function of hematopoietic cells (Kratz et al., 2007; Zhu et al., 2011), the chemotherapy-induced anemia could be related to the development of immunosuppression and cytotoxicity on hematopoietic cells. After the drug administration, the expression of IL-2 and SHP2 was increased, suggesting that FEJ and SPN could make a positive effect on enhancing the T cell immunity and reducing the cytotoxicity. Meanwhile, it was reported that SHP2 directly mediates many cytokines and growth factor receptors like GM-CSF and EPO receptors (Myers et al., 1998). The increased mRNA expression of GM-CSF and EPOR shown in **Figure 3** could be partly attributed to the up-regulation role of SHP2 intervened by SPN. The role of drugs on another important signal transducer and activator of transcription, STAT 1, was investigated by Western blot and RT-qPCR. As shown in **Figure 6B**, the chemotherapy-induced the expression of STAT 1 mRNA, which was decreased in a dose-dependent way when treated with SPN. However, the activation of STAT1 protein level (**Figure 5**) was inconsistent with its mRNA expression. This might be due to that the post-transcriptional RNA and the binding sites of regulatory proteins changed the mode of translation and then affected the expression of proteins. Such phenomenon was also investigated in previous studies (Saunders et al., 2007; Nicoloso et al., 2016). Another possible explanation may be that the post-translational modification led to a decrease in the stability or half-life of the protein. Also, the activity of JAK1 and its phosphorylated form, the enzymes essential for the receptor signaling located in the upstream of STAT1, was investigated. According to the results, the treatment of drugs made no significant effect on the expression of JAK1, whereas the expression of p-JAK1 was increased after the chemotherapy and then decreased after treating with FEJ and H-SPN. It suggested that FEJ and H-SPN might regulate the hematinic function through phosphorylating JAK1.

**Figure 8:**
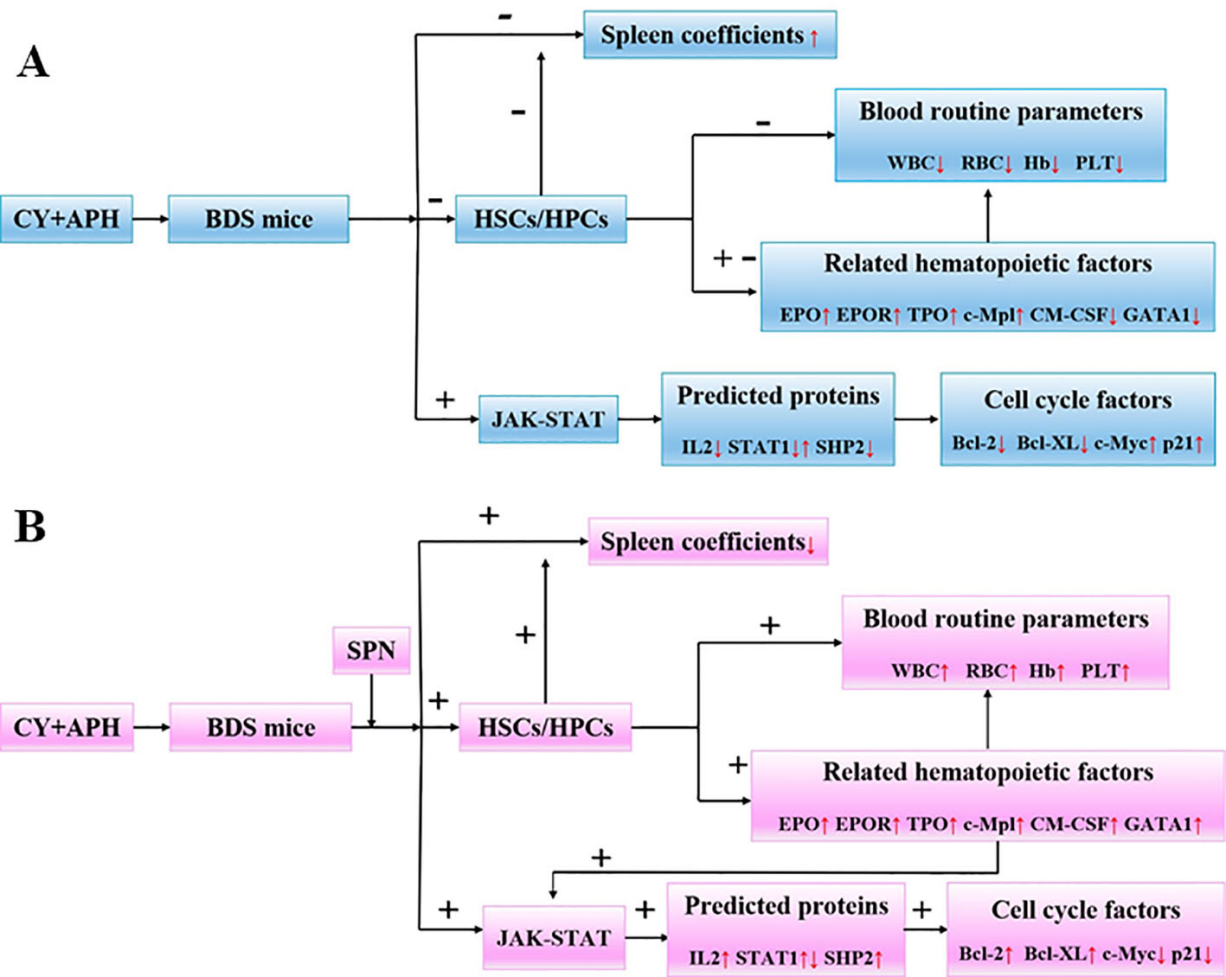
**(A)** Changes of body parameters and cytokines in BDS mice compared to the control group. **(B)** Changes of body parameters and cytokines in SPN administration groups mice compared to the model group. IL-2, interleukin-2; JAK1, janus kinase-1; SHP2, tyrosine-protein phosphatase non-receptor type 11; GRB, growth factor receptor-bound protein; SOS, guanine nucleotide exchange factor; STAT, signal transducer and activator of transcription; Bcl-2, B-cell lymphoma-2; Bcl-XL, Bcl-2 like 1; c-Myc, v-myc avian myelocytomatosis viral oncogene homolog; p21, cki.

**Figure 9:**
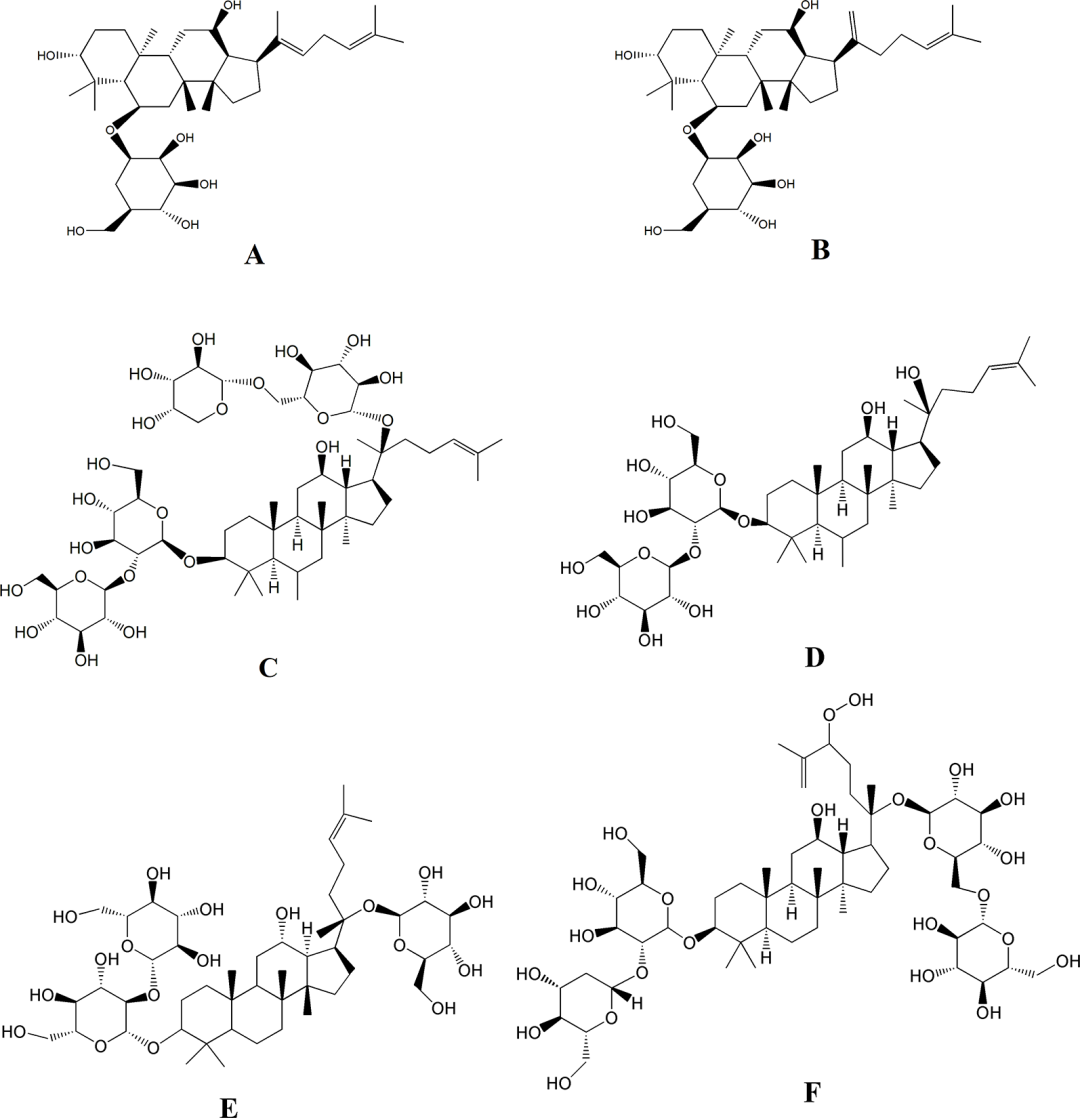
The six compounds corresponding to the three targets involved in the JAK-STAT signal pathway related to the treatment of BDS with SPN. **(A)** Ginsenosides Rh_4_, **(B)** ginsenosides Rk_3_, **(C)** ginsenosides Rb_2_, **(D)** ginsenosides 20(*R*)-Rg_3_, **(E)** ginsenosides Rd, and **(F)** notoginsenoside C.

Besides the above targets involved in the pathway (**Figure 8**), network pharmacology also provides the chemical information related to the efficacy of SPN, which is therefore beneficial to uncovering the interactions between active constituents in SPN and the complex syndrome systems (Tao et al., 2013; Sheng et al., 2014). By the analysis of network pharmacology, six compounds in SPN were predicted to be the anti-anemia ones corresponding to the three targets involved in the JAK-STAT signal pathway, which were ginsenosides Rh_4_, Rk_3_, Rb_2_, 20(*R*)-Rg_3_, Rd, and notoginsenoside C (**Figure 9**). Differences in ginsenoside structure which include the type, position, and number of sugar moieties attached by a glycosidic bond can characteristically influence the biological responses (Teng et al., 2017; Chen et al., 2019). Among them, 20(*R*)-Rg_3_, Rh_4_, and Rk_3_ showed increased levels along with the duration of steaming and had a key role in the activities of SPN in our previous study (Xiong et al., 2017b). Zhou et al. (2016) reported that Rg_3_ helped repair blood disorders such as anemia, leukopenia, and thrombocytopenia. 20(*R*)-Rg_3_ could also stimulated the production of IL-2 and enhance the cellular immunity in tumor-bearing mice (Wu et al., 2014), which was consistent with our result of IL-2 expression. Ginsenosides Rh_4_ and Rk_3_ were considered to be new therapeutic drugs for treating anemia since Rk_3_ promoted bone marrow and extramedullary hematopoiesis while Rh_4_ promoted extramedullary hematopoiesis (Wei et al., 2018). Different from Rg_3_, Rb_2_ and Rd have an addition of one glucose moiety and two glucose moieties attached at C-20 position. Rb_2_ could protect the hematopoietic system from the irradiation-caused injury and promote the synthesis of serum protein and DNA of marrow cells (Lee et al., 2011; Zhang et al., 2017). Ginsenosides Rd could regulate the expression of Bcl-2 (Wang et al., 2013). Notoginsenside C was only mentioned in a few reports (Yoshikawa et al., 2001; Chen et al., 2010) which could have the anti-tumor and immunological adjuvant activity. In further studies, the effects of those constituents and related mechanism will be investigated and confirmed.

Bcl-2, Bcl-XL, c-Myc, and p21 are cell cycle-related factors of JAK1-STAT1. SPN could act on the JAK1-STAT1 signaling pathway and simultaneously affect the downstream cytokines, improving the cell apoptosis of mice with BDS. According to **Figure 7A**, the expression of Bcl-2 was up-regulated along with the dose increase of SPN, which could maintain the survival and proliferation of erythroid progenitor cells when GATA-1 was reduced in the model of anemia (**Figure 3F**) (Kapur and Zhang, 2001). Another anti-apoptotic protein of Bcl-XL was also up-regulated when treating with SPN (**Figure 7B**), which might be due to the mediation of GATA1, EPO and TPO through the JAK/STATs pathway, supporting the survival of hematopoietic cells (Chiba et al., 1991; Kitajima, 2006). c-Myc and p21, the expression of which were often increased in hematological malignancies caused by BDS or DNA damage, were both down-regulated significantly after giving SPN (**Figure 7C**), suggesting that SPN might attenuate anemia by inhibiting the arrest of cell cycle because of DNA damage. Therefore, the expression of various hematopoietic factors could be closely related to the cell apoptosis. This is consistent with the report of Ćmielová and Řezáčová, (2011).

## Conclusions

The hematopoietic effect and mechanism of SPN on mice with BDS was studied at three levels. Firstly, SPN reverted the decrease in the number of peripheral blood cells and prevented the enlargement of the extramedullary hematopoietic organ of spleen. Secondly, SPN promoted the expression of related hematopoietic cytokines including EPO, EPOR, TPO, c-Mpl, GM-CSF, and GATA-1 in bone marrow nucleated cells of mice. Thirdly, SPN was involved in the activation of JAK-STAT signaling pathway, through regulating the targets expression of IL-2, p-JAK, STAT1, and SHP, and also the downstream cell cycle factors of Bcl-2, Bcl-XL, c-Myc, and p21 (**Figure 8**). SPN could influence the cell cycle and possibly the apoptosis process, enabling the damaged cells to obtain a favorable survival condition in a blood deficiency environment. The present study provides further guidance for developments in the clinical use and as well as provides clues for drug development of SPN.

## Data Availability Statement

All datasets generated for this study are included in the article/**Supplementary Material**.

## Ethics Statement

The animal study was reviewed and approved by the Experimental Animal Welfare and Ethics Committee, Kunming University of Science and Technology.

## Author Contributions

YX supervised the project and participated in the design of the study. ZZ performed the experiments and statistical analysis as well as the writing of the paper. YY gave experimental guidance. MG provided the revision and correction works. YZ, YH, CW, and XC collected and processed samples. BD and MW guided the design and modified the manuscript.

## Funding

This work was supported by the National Natural Science Foundation of China (81660661), the Yunnan Applied Basic Research Project (2016FD040), and the National Research Program of China (2017YFC02503).

## Conflict of Interest

Author BD was employed by company Fytagoras BV.

The remaining authors declare that the research was conducted in the absence of any commercial or financial relationships that could be construed as a potential conflict of interest.

## Abbreviations

APH, acetyl phenylhydrazine; Bcl-2, B-cell lymphoma-2; BDS, blood deficiency syndrome; c-Mpl, thrombopoietin receptor; c-Myc, v-myc avian myelocytomatosis viral oncogene homolog; CY, cyclophosphamide; EPO, erythropoietin; EPOR, erythropoietin receptor; FEJ, *Fufang E'jiao Jiang;* GM-CSF, granulocyte-macrophage colony-stimulating factor; Hb, hemoglobin; IL-2, interleukin-2; JAK-STAT, janus kinase-signal transducer and activator of transcription; KEGG, Kyoto Encyclopedia of Genes and Genomes; PN, *Panax notoginseng;* PLT, platelet; RBC, red blood cell; PT-qPCR, real-time quantitative polymerase chain reaction; SHP-2, tyrosine-protein phosphatase non-receptor type 11; SPN, steamed *Panax notoginseng;* TPO, thrombopoietin; WBC, white blood cell.
