# Supplementary material for: Steamed Panax notoginseng Attenuates Anemia in Mice With Blood Deficiency Syndrome via Regulating Hematopoietic Factors and JAK-STAT Pathway
Source: Front Pharmacol. 2020 Jan 21;10:1578. doi: 10.3389/fphar.2019.01578 (PMC6985777; doi:10.3389/fphar.2019.01578)
Supplement: Supplementary file 1 [file DataSheet_1.docx]

**TABLE S1 Results of power analysis to determine the animal sample size**

| **Power** | ***n*** | ***k*** | ***N*** | **Alpha** | **Beta** | **Standard deviation of means** | **Standard deviation** | **Effect size** |
| --- | --- | --- | --- | --- | --- | --- | --- | --- |
| 0.92324 | 10 | 6 | 60 | 0.05000 | 0.07676 | 5.15 | 9.00 | 0.5726 |
| 0.92324 | 10 | 6 | 60 | 0.05000 | 0.07676 | 5.15 | 9.00 | 0.5726 |
| 0.92324 | 10 | 6 | 60 | 0.05000 | 0.07676 | 5.15 | 9.00 | 0.5726 |
| 0.92324 | 10 | 6 | 60 | 0.05000 | 0.07676 | 5.15 | 9.00 | 0.5726 |
| 0.92324 | 10 | 6 | 60 | 0.05000 | 0.07676 | 5.15 | 9.00 | 0.5726 |
| 0.92324 | 10 | 6 | 60 | 0.05000 | 0.07676 | 5.15 | 9.00 | 0.5726 |

Power represents the probability of rejecting a false null hypothesis.

*n* represents the average group sample size.

*k* represents the number of groups.

*N* represents the total sample size of all groups combined.

Alpha represents the probability of rejecting a true null hypothesis.

Beta represents the probability of accepting a false null hypothesis.

Standard deviation of means represents the standard deviation of the group means under the alternative hypothesis.

Standard deviation represents the within group standard deviation.

Effect size represents the ratio of standard deviation of means to standard deviation.


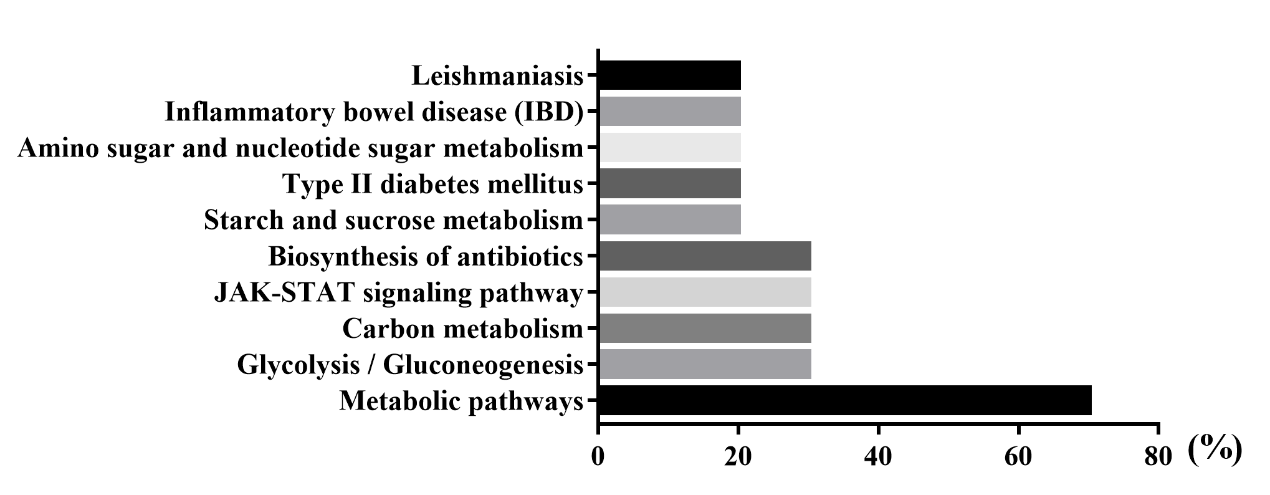


**FIGURE S1** 10 Kyoto Encyclopedia of Genes and Genomes (KEGG) pathways obtained by the pathway-enrichment analysis. Gene and protein targets associated with the disease of anemia were collected from the Online Mendelian Inheritance in Man (OMIM) database (Amberger et al., 2015). DAVID Functional Annotation Bioinformatics Microarray Analysis (https://david.ncifcrf.gov/) was performed (Dennis et al., 2013) for the pathway enrichment analysis. *X*-axis represents the percentage of targets involved in the pathway. *Y*-axis represents the name of pathway (Xiong et al., 2018).


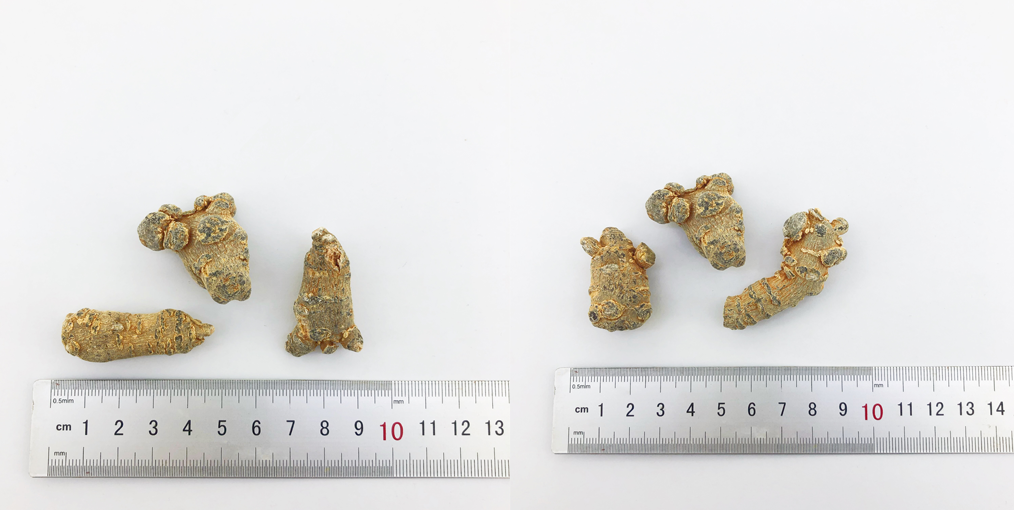


**FIGURE S2** *Panax notoginseng* specimen (No. WSPN15101) deposited in Yunnan Key Laboratory of *Panax notoginseng*, Kunming University of Science and Technology.


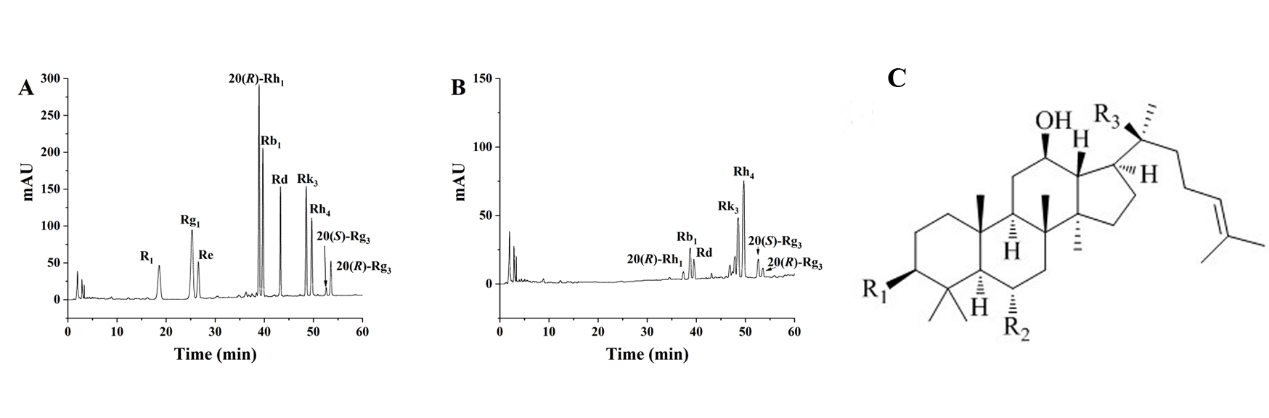


| Elution sequence | Compound | Content (%) | R_1_ | R_2_ | R_3_ |
| --- | --- | --- | --- | --- | --- |
| 1 | Notoginsenoside R_1_ | 0 | -OH | -OGlc[2→1]xyl | -OGlc |
| 2 | Ginsenoside Rg_1_ | 0 | -OH | -OGlc | -OGlc |
| 3 | Ginsenoside Re | 0 | -OH | -OGlc[2→1]Rha | -OGlc |
| 4 | Ginsenoside Rh_1_ | 0.04 | -OH | -OGlc | -OH |
| 5 | Ginsenoside Rb_1_ | 0.52 | -OGlc[2→1]Glc | -H | -OGlc[6→1]Glc |
| 6 | Ginsenoside Rd | 0.032 | -OGlc[2→1]Glc | -H | -OGlc |
| 7 | Ginsenoside Rk_3_ | 1.66 | -OH | -OGlc | Dehydration at C-20 |
| 8 | Ginsenoside Rh_4_ | 2.73 | -OGlc[2→1]Glc | -H | Dehydration at C-20 |
| 9 | Ginsenoside Rg_3_ | 0.78 | -OGlc[2→1]Glc | -H | -OH |
| 10 | Ginsenoside Rg_3_ | 0.48 | -OGlc[2→1]Glc | -H | -OH |

**FIGURE S3** HPLC chromatograms of (A) the mixed standards solution and (B) SPN sample as well as (**C)** chemical structures of saponins presented in SPN (Xiong et al., 2017). HPLC separation was performed done on an on a Vision HT C18 column (250 mm×4.6 mm, 5μm) at a temperature of 30℃. The mobile phase consisting of A (ultra pure water) and B (MeCN) was used at a flow rate of 1.0 ml/min as the following gradient mode: 0-20 min, 80% A; 20-45 min, 54% A; 45-55 min, 45% A; 55-60 min, 45% A; 60-65 min, 100% B; 65-70 min, 80% A; 70-90 min, 80% A. The detection wavelength was set at 203 nm and the injection column was set at 10 μl. HPLC, high performance liquid chromatography; SPN, steamed *Panax notoginseng*.

**Reference**

Amberger, J. S., Bocchini, C. A., Schiettecatte, F., Scott, A. F., and Hamosh, A. (2015). OMIM.org: Online Mendelian Inheritance in Man (OMIM®), an online catalog of human genes and genetic disorders. *Nucleic. Acids. Res.* 43(1), 789–798. Doi: 10.1093/nar/gku1205.

Dennis, G., Sherman, B. T., Hosack, D. A., Yang, J., Gao, W., Lane, H. C., et al. (2013). DAVID: Database for annotation, visualization, and integrated discovery. *Genome. Biol*. 4(9), R60. Doi: 10.1186/gb-2003-4-9-r60.

Xiong Y., Hu, Y. P., Chen, L. J., Zhang, Z. j., Zhang, Y. M., Niu, M., et al. (2018). Unveiling active constituents and potential targets related to the hematinic effect of steamed *Panax notoginseng* using network pharmacology coupled with multivariate. *Front. Pharmacol*. 9, 1514. Doi: 10.3389/fphar.2018.01514.

Xiong, Y., Chen, L. J., Man, J. H., Hu, Y. P., and Cui, X. M. (2017). Chemical and bioactive comparison of *Panax notoginseng* root and rhizome in raw and steamed forms. *J. Ginseng Res*. 1-9. Doi: 10.1016/j.jgr.2017.11.004.
